# Assessment of the Level of Anxiety for COVID-19 Vaccinations

**DOI:** 10.3390/vaccines10060915

**Published:** 2022-06-09

**Authors:** Marcin Świerad, Ilona Świerad, Robert Szydło, Grzegorz Honisz, Mariusz Gąsior, Zbigniew Kalarus, Krzysztof Dyrbuś

**Affiliations:** 1 Department of Cardiology, Congenital Heart Diseases and Electrotherapy, Silesian Center for Heart Diseases in Zabrze, 41-800 Zabrze, Poland; marcinswierad@gmail.com (M.Ś.); g.honisz@sccs.pl (G.H.); z.kalarus@sccs.pl (Z.K.); 2 Psychological and Pedagogical Counseling Center No. 3 in Katowice, 40-096 Katowice, Poland; ilonaswierad@gmail.com; 3 Department of Labor Resource Management, Cracow University of Economics, 31-510 Kraków, Poland; 4 3rd Department of Cardiology, Faculty of Medical Sciences in Zabrze, Medical University of Silesia, 40-055 Katowice, Poland; m.gasior@sccs.pl (M.G.); k.dyrbus@sccs.pl (K.D.)

**Keywords:** vaccinating, COVID-19, anxiety state, anxiety trait, decision-making

## Abstract

Research published especially in the last decade indicates the influence of anxiety on the human decision-making process. This study analyzes the anxiety among individuals who decided to undergo vaccinations for COVID-19. The study assesses that the level of education, especially medical education, age, and gender, had an influence on the level of anxiety in terms of vaccination situations. The STAI self-assessment questionnaire was used. The study was conducted anonymously using the paper-pencil method during two rounds of vaccination; therefore, the respondent sample included mainly medical personnel and elderly people. A total of 898 questionnaires were issued. Age did not affect the trait and state of anxiety, but highly educated people tested during vaccination had a lower anxiety level. Gender had no influence on the trait but did influence the state of anxiety. Overall, women were the group that exhibited a higher level of anxiety than men. Nurses were particularly vulnerable to the negative effects of situational medication in this group.

## 1. Introduction

A neuro-economic approach combining scientific methods originating in medicine, management, economics, and psychology is becoming more and more popular. In recent years, there has been research linking anxiety with management and economic decision-making processes. The present research paper is the result of such an interdisciplinary approach.

Anxiety is a negative emotional state related to predicting a threat from inside or outside the body, manifested as anxiety, a feeling of tension, or danger [1,2]. Unlike fear, it is an internal process not related to a direct threat or pain [3]. The anxiety experienced by a person has a strong, negative impact on cognitive processes: it distorts attention, limits memory, impedes or prevents both association and concentration, hinders decision-making, weakens thinking, causes an inadequate assessment of one’s abilities, and overwhelms and paralyzes [2].

The COVID-19 pandemic can be treated as a crisis, understood as a unique challenge that brings both threats and opportunities. It has an undeniable impact on our lives in every dimension—on a macro and micro scale, from entire economic systems, countries, and enterprises to each of the individual human beings that it affects. National economies require the fastest possible stabilization, and an epidemic situation means the necessity to overcome the virus, which can be obtained by mass vaccination. However, there is no widespread readiness to vaccinate among Polish citizens. Research by the Public Opinion Research Center [4] shows that half of the respondents did not show any intention to vaccinate. Most of these people were concerned about the side effects of the vaccine. Their attitude may be understood by studies conducted in recent years noticing the relationship between anxiety and decision-making.

The undertaken studies assessed the level of anxiety in people deciding to receive the COVID-19 vaccine and whether it depends on other variables such as education, age, and gender. It also considers the modality and its change with the first and second dose of vaccination.

### 1.1. Getting Vaccinated against COVID-19 as a Decision-Making Process

Already in 2018, the Polish Pharmaceutical Chamber [5], in the face of ongoing public discussion in the Parliament about a law to abolish the compulsory vaccination of children, announced in the media its opinion in a statement that called for “a cessation of activities related to the liquidation of coercion vaccinations in Poland, “arguing that” … There is no more effective method of protection against infectious diseases than vaccination. … The legitimacy and obligation of vaccinations should not raise any doubts, because rigorous scientific research, which confirms the quality and effectiveness of vaccines, is also a guarantee of their safety. The facts clearly show the need for vaccinations and the certainty of their administration. Decisions not to vaccinate are based on a lack of awareness and are not in line with current science and medical knowledge. Public awareness of vaccination is negligible, and single cases (in the scale of the entire population), publicized and exaggerated by anti-vaccination movements, cause increased concerns and, consequently, the decision not to vaccinate.” In the first days of December 2020, about a year after the pandemic outbreak, the Reuters agency reported that over 1.5 million people lost their lives due to COVID-19. This was very vividly presented: on average, one person dies every nine seconds from the coronavirus. Currently, it has already resulted in over 5.2 million deaths [6]. In Poland, the highest increases in infections and deaths due to COVID-19 occurred in November 2020 and April 2021 [6]. It was the highest recorded monthly number of deaths in five years. In the first week of November, the number of deaths was 112% higher than in the corresponding period a year earlier.

Despite these reports, surveys by the Public Opinion Research Center [4], conducted in November 2020, indicated that if the vaccine were available, only one in three respondents would get vaccinated against COVID-19. The results indicated that the attitude to vaccination depends on age, gender, and wealth. The CBOS report showed that “respondents at least 45 years old more often declare their intention to get vaccinated than younger respondents, and most often such intention is expressed by the eldest aged 65 and more (49%, including 26% expressing it strongly)”. The survey showed that men more often than women declare their intention to get vaccinated (41% compared to 31%). In addition, respondents from households with higher per capita incomes expressed their willingness to vaccinate themselves more often (45% of those with an income of PLN 3000 and more).

These studies link the attitude to vaccination with one’s health and the epidemic itself. The vast majority of the declared reluctance to vaccinate against COVID-19 was the concern about the potential side effects of the new vaccine (69%). Concerns about a lack of vaccine effectiveness were mentioned less frequently (25%). Almost one in three people who do not declare their intention to vaccinate do not want to be vaccinated because they avoid vaccinations altogether (30%). Less frequent arguments were made that COVID-19 is not a severe disease (11%), that the vaccine may be too expensive (6%), and that SARS-CoV-2 has already infected them (4%). Concerns about the side effects that a new vaccine may cause are more often reported by women than men (73% versus 65%), younger respondents under 45 (71–77%, depending on the category), and those who are better educated (76%)—primarily graduates of universities and respondents from households with a per capita income of at least PLN 3000 (82%).

Fears related to the lack of vaccine effectiveness are more often expressed by residents of large (33%) and the largest cities (37%), respondents who are better educated (32% of university graduates), respondents aged 45–54 (37%), and respondents from households with a per capita income of at least PLN 3000 (39%). On the other hand, avoiding vaccinations altogether is more often declared by older respondents, most often aged 55–64 (46%), living in smaller towns and villages (37% of rural residents), with primary vocational education (41%), and with per capita income in the household ranging from PLN 1000 to PLN 1999 (32–33% depending on the category).

Compared to the results of studies from previous years, also carried out in the first half of November, the percentage of respondents who were vaccinated against influenza in a given season has practically remained the same (6%), but the percentage of respondents declaring an intention to vaccinate against influenza has increased (from 7% to 14% compared to 2016). Research has shown that one in fourteen respondents declared that they had vaccinated against influenza every season (7%) in the last five years (7%), and every ninth had done it irregularly (11%). So far, people declaring their intention to vaccinate against COVID-19 are in the minority. Slightly more than every third adult Pole would like to get vaccinated against the disease if a vaccine was available, and nearly half do not intend to do so [4].

In June 2021, in a CBOS study, 18% of respondents wanted to be vaccinated, and 26% of respondents still did not want to be vaccinated. Thus, there was a decrease in people willing to be vaccinated (from 33% to 18%) and a constant percentage of people who did not declare their willingness to be vaccinated. However, this is related to 52% of people declaring vaccination with at least one dose [7].

It is important to state that clinical manifestations may occur and have a high impact on the decision-making process. They are presented in Table 1.

### 1.2. Anxiety as a Behavior Regulator

Cattell and Scheier [11] introduced the concepts of the state and trait of anxiety to psychology. They introduced this division based on mathematical analyses of the collected empirical material. Cattell based his distinction between anxiety as a state and trait on the length of time anxiety reactions lasted and their repetition. In this approach, the state of anxiety is understood as a group of specific anxiety reactions lasting some time. On the other hand, in the trait of anxiety, a group of the same anxiety reactions that occur relatively constantly for each individual can be assessed using multiple tests repeated every few weeks or months [12].

Spielberger and colleagues [13] also understand anxiety in two ways: a state felt in a specific situation and a relatively constant personality trait. Anxiety as a state is characterized by subjective feelings of fear and tension accompanied by associated excitement of the nervous system. A characteristic feature of this type of fear is its high variability under the influence of threatening factors [2]. Anxiety as a trait [13] denotes an acquired behavioral predisposition that makes an individual susceptible to perceiving an extensive range of objectively non-threatening stimuli as dangerous and reacting to them with a state of anxiety that is unreasonably high concerning the magnitude of the objective danger. Fear as a trait has a learned character [14] with relative stability in this understanding. It is a disposition of an anxious way of reacting.

The concept of anxiety presented by psychologists Endler and Parker [15] is, in a sense, an extension and supplement to Spielberger’s proposition. According to this concept, the state of anxiety is a function of the interaction of personality and situational factors. According to Endler, there are three types of anxiety-producing situations: ego-threatening situations (interpersonal situations), hazardous physical situations, and ambiguous and unclear situations.

Anxiety plays an essential role in the adaptation process of the human body, both as a factor stimulating the response to stress and as a precursor to further stress reactions. Formański [16] believes that anxiety and fear fulfill adaptive functions as they prepare the body to defend itself in the face of a threat. A low level of anxiety is of great importance as a threat signal that precedes or accompanies the adjustment process. With a high level of anxiety, the balance is so disturbed that psychological processes and somatic functions become disorganized [17]. A person’s adaptation to a new situation is accompanied by emotions of various shades and strengths [18]. While positive emotions favor constructive actions, motivation to act, and development, negative emotions can inhibit actions and lead to withdrawal [14]. Among the emotions experienced at that time, fear most strongly influenced human activity and creativity [19].

However, acting in conditions of increased anxiety has a destructive effect on the functioning of a person—cognitive exhaustion, somatic diseases, changes in social functioning, and difficulties in making adequate decisions [14]. The decision to undergo COVID-19 vaccination is subject to the same mechanisms and decision-making processes as other choices [20]. Anxiety is one of the essential variables influencing human health and the decision-making process itself. In extreme cases, anxiety has a negative impact on making decisions and even avoiding them. Decisiveness in a situation of choice is a strategy for coping with stressful situations [21]. Decisiveness is a mechanism for dealing with a natural or potential identity threat in ambiguous situations because it comes down to doing nothing. The impact of anxiety on the decision-making process, including decisions in the field of management or economics, may have a biological basis, as indicated, among other things, by research by Hartley and Phelps [22] from the University of New York in the USA. The results of their work devoted to this show that anxiety, intense fear, and terror that accompany the disease directly impact daily decision-making processes. In their work, they pointed to the partial overlapping of neural systems, which are the mediators of fear and anxiety, with those indicated in research on economic decision-making. The circuits that include the amygdala, insular cortex, and prefrontal cortex are involved in tasks related to uncertainty or loss. The amygdala is a crucial area of the brain that regulates fear and anxiety levels, and the prefrontal cortex is critical to fear control. It can be concluded that fear and anxiety influence choices. A team of researchers from the University of New York and the University of Beijing [23] found that stress reduces our ability to respond to new threats and anticipate new dangers. When we are under stress, we pay less attention to environmental changes, which exposes us to new threats. Too high a level of anxiety in people may have a negative impact on the perception and processing of the information obtained [24] and thus may hinder making logical health-related decisions. The literature on the subject provides numerous examples proving that a high level of anxiety may even make it impossible to make any decisions [21,25].

It is important to emphasize that there are various factors that may differentiate anxiety as a state and trait within the group of medical professionals. The group was chosen because of two factors. First of all, medical professionals are on the first line in the fight with COVID-19 and any other pandemic, and the vaccination of this group is crucial for the whole medical system. Medical professionals as experts are being used in various communication methods with society, and as experts, they may set an example for other members of the society. However, this group is not homogeneous. There are people of different ages, genders, and education levels among the medical staff. Age is a differentiating factor for anxiety state and trait [26,27] as well as in the preventive behavior connected with COIVD-19 [28]. Other articles confirm the importance of gender differences [29,30], as well as education [31,32], for anxiety understood as a state and trait. The studies mentioned above allow us to predict the relationship between the level of anxiety and the decision to vaccinate for COVID-19. Research on the connection between anxiety and the decision to vaccinate is fundamental in the context of subsequent waves of the disease and designing campaigns and activities to encourage vaccination. The results may be of particular interest to employers and vaccine program services and organizations.

As part of the exploratory research, two research questions were asked:

1. Whether the level of anxiety as a state and trait is related to vaccination decisions among health care workers.
2. How should health care professionals and society be influenced to overcome the anxiety toward vaccination?

## 2. Materials and Methods

In order to answer the research questions, it was decided to link the level of state and trait of anxiety of people who decided to vaccinate with other psychosocial variables such as age, gender, education (especially medical education), and occupation of people vaccinated against COVID–19.

To verify the level of state and trait of anxiety, the STAI (State-Trait Anxiety Inventory): Self-Assessment Questionnaire was used. It was translated into Polish by Spielberger, Strelau, Tysarczyk and Wrześniewski [2]. It is an inventory of the state and trait of anxiety. In the scale created by Spielberger and colleagues, anxiety is examined both as a state (felt in a specific situation) and as a trait (relatively constant for a given individual personality trait). The results were calculated according to the recommendations of the questionnaire. STAI questionnaire is considered a C-level test, which states that only psychologists, after master’s studies, can use it. Two psychologists were responsible for gathering and calculating the results in the research teams: Ilona Świerad and Robert Szydło. The calculation was performed according to the strict instructions in the guide book for STAI users [13].

The respondents also completed the questionnaire in terms of socio-geographic variables. They entered their age numerically, declared their gender (male/female), specified their education (they entered freely, and then the researchers classified them as primary, secondary, vocational, post-secondary, and higher) and occupation (they entered it independently, and then it was qualified by the researchers as a medical or non-medical profession).

Statistics were made based on descriptive measures and tests:

1. Shapiro–Wilk test to verify the normality of the distribution
2. Measure of central tendency—mean (M), median (Md), quartiles (Q1, Q2, Q3), and Srandared deviation (SD)
3. Diffusion measures (standard deviation, minimum and maximum values)
4. Rho-Spearman test examining the correlation between pairs of variables
5. U Mann–Whitney (U) investigating the difference between two medians

The research was anonymous; each respondent could resign from the study at any stage. A total of 595 people were included in the study. The mean age of the respondents was Md = 44, with Q1 = 33, Q2 = 44, and Q3 = 54. The youngest person in the study was 20 years old, and the oldest was 85 years old.

A total of 898 questionnaires were issued, including 359 questionnaires for the first vaccination round and 236 questionnaires for the second round of vaccination. Unfortunately, 303 questionnaires have been erased from the research sample because of either abandonment of the study or random filling of the questionnaire. The study did not consider sheets that were not correctly completed, including those that were incomplete. The majority of the surveyed people were medical professionals. Moreover, there were 91 doctors and 164 nurses among them, as well as 154 other medical professionals, such as paramedics, laboratory technicians, and medical analysts. The research was carried out using the paper-pencil method, which was forced for organizational reasons. Table 2 provides the information about the description of each criterion.

## 3. Results

In order to describe the research sample and meet the goals of the research, various statistical analyses were performed. Due to the high number of tests, only the most important ones are presented in the research paper. Table 3 shows the results of the conducted research in an aggregated form.

The results were assessed for normality using the Shapiro–Wilk test. The distribution of the study group is not close to normal in the context of the state W (595) = 0.953, *p* < 0.001 and the anxiety trait W (595) = 0.939, *p* < 0.001. Therefore, in further statistical calculations, non-parametric tests were used. In the analyzed research group, the correlation between the trait and the state of anxiety was rs = 0.600, *p* < 0.001, N = 595, which confirms the reliability of the research. In the analyzed research group, there is a large overrepresentation of people with a low level of anxiety trait and a high overrepresentation of people with a low anxiety state concerning the population. 

During the analysis, it was not found that age was related to the state (rs = 0.033, *p* = 0.211, N = 595) or the trait of anxiety (rs = −0.063, *p* = 0.062, N = 595) in the population. Similar results were observed in the group of medical professionals. However, when examining the doctors themselves, a significant negative correlation between age and the state of anxiety was found (rs = −0.179, *p* = 0.045, N = 91); however, this is a weak correlation. For the group of nurses, the results were statistically insignificant.

In the study group, it was shown that women show a greater state of anxiety than men: U (women = 448, men = 147) = 25,068.5, z = −4.393, *p* < 0.001. For medical professionals, the same U results were observed: (women = 312, men = 97) = 10,048.5, z = −4.055, *p* < 0.001. In the group of doctors, the results turned out to be statistically insignificant, and there was no nurse (men) among the respondents—all the nurses were women.

In the general research group, it was shown that people with secondary education have a statistically significantly higher level of anxiety (Md = 5) than people with higher education (Md = 4): U (secondary = 128, higher = 405) = 22,549, z = −2.244, *p* = 0.025. Their results of comparison between other education groups were statistically insignificant (*p* > 0.05).

It is worth noting a statistically significantly higher level of anxiety, U (medical = 409, non-medical = 186) = 33,905.5, z = −2.149, *p* = 0.032, in medical professionals (M = 4.27, Md = 4) compared to people in other professions (M = 3.84, Md = 4). A similar relationship can be observed in nurses but not in doctors. Nurses (Md = 5) have a statistically significant level of anxiety, U (nurse = 164, others = 431) = 29,461, z = −3.173, *p* = 0.002, than people working in other professions (Md = 4).

The first and second dose results in both anxiety as a state and trait were as follow: For state U (first dose = 359, second dose = 236) = 41,838.5, z = −0.258, *p* = 0.796 and for trait U (first dose = 359, second dose = 236) = 41,942, z = −0.208, *p* = 0.835.

## 4. Discussion

In this research group, there was a positive correlation between the trait and the state of anxiety. This may indicate a significant stressful impact of the pandemic and peri-vaccination situation. The activation of internal anxiety dispositions indicates that the vaccinees most likely perceived this situation as characterized by a high intensity of risk factors. According to Spielberger, anxiety as a trait is an indicator of the ability of people to react in stressful situations [33,34] and is revealed in people with a strong disposition to anxiety in situations in which they are not sure of its location [33]. Therefore, it can be concluded that in those strongly conditioned to experience anxiety, arousing anxiety as a state proves that they perceived the pandemic situation and the related vaccinations as a strong threat. Polish research results also confirm this line of reasoning. Sosnowski and Wrześniewski [35] also held that people with a high intensity of the anxiety trait react with it in situations with an appropriate intensity of threatening factors and do not have to manifest it continuously. Similarly, Badura-Brzoza, Matysiakiewicz, and Piegza [36] associated higher values of state anxiety in the group of patients treated surgically by amputation of a limb with the difficulties and challenges of the new situation in which they found themselves and with the change in their body image.

The research was carried out on a research group that had the right to vaccinate against COVID-19 as the so-called “group zero.” It consisted of medical professionals and elderly people over 70. The obtained results did not show any signs of a normal distribution. The results obtained can be explained by the specificity of the groups selected for vaccination. Vaccination was not compulsory or systemically forced. Specific individuals were granted the right to vaccinate but were subject to it voluntarily, and there were no sanctions, restrictions, or other forms of pressure associated with vaccination. They carried out a self-process of inference and profit-and-loss analysis, which enabled them to vaccinate for COVID-19. Therefore, due to their relatively low level of anxiety, they decided to vaccinate. For eligible volunteers, there was a lack of distribution under normal duress and a significant overrepresentation of people with a low level of trait and anxiety.

Age was not related to the state or trait of anxiety. Although, in the case of physicians, a statistically significant negative correlation between age and anxiety level was demonstrated, the strength of this correlation (rs = −0.179) may be treated as negligible and will not be subject to further discussion.

Women are characterized by a higher state of anxiety in emergencies, which is confirmed by research [2]. Prior to vaccination, university graduates responded less anxiously. Other studies confirm this regularity. Lewicka et al. [37] indicate a relationship between education and the level of anxiety in the studies mentioned above on patients undergoing surgery for gynecological reasons: the severity of state anxiety and trait anxiety was significantly greater in women with primary or vocational education than with secondary and higher education. The influence of education on anxiety is confirmed in the analysis of the results in the group of medical professionals: the higher the level of education, the lower the level of anxiety in stressful situations. The study by Sjoling et al. [38] outlines the role of the patient in the prevention of pain in the postoperative period and in returning to full fitness in the postoperative period. This suggests the legitimacy of providing in-depth information, understandable to patients regardless of their education and extending its typically informative scope, with additional information emphasizing the patient’s causative power in improving their situation. The lack of exhaustive information correlates with the dissatisfaction of the doctor-patient relationship. The information package should include factual information about vaccination, its nature, and methods of operation, as well as possible choices, a risk assessment, and possible complications. It should sensitize and present the possibilities of improving one’s health condition and appropriate interactions at the time of their occurrence [39]. It is important to state that the results of education impact on the anxiety level are considered within the medical professions, not in the comparison of medical and other professions. This is due to the specific question. The question is, What is the source of fear among medical staff? It seems that the exposure to suffering and the feeling of constant threat they face daily have resulted in an increased state of anxiety. Because of that constant exposure, it is impossible to compare the education level of medical professionals with other professions.

## 5. Conclusions

Based on the presented research results, specific actions can be proposed, both in the context of society and strictly for medical personnel, in order to reduce the feeling of anxiety associated with vaccination as a state and thus facilitate the decision to vaccinate and manage the anxiety manifestations.

For managers of hospitals and health care units:

1. Direct actions toward junior doctors
2. Focus on working with women employed in health care units
3. Provide access to the broadest possible collection of information on immunization
4. Present the results of the latest research

In order to conduct social campaigns that will prevent the anxiety manifestations:

1. Target all age groups within the knowledge-based communication
2. Focus on reporting reliable research results
3. Limit activities focused on the emotionality and social structure of the country
4. Enhance information activities for people between vaccination series so that people do not drop out of the second or third dose of the vaccine

The conducted research is subject to limitations. The most important of these includes the research sample, which was limited to seniors and medical personnel, the inequality of individual respondents, and a high level of withdrawal from participation in the study. However, these limitations are related to the nature of the vaccination process. In the future, researchers plan to continue research on the state and trait of anxiety in various aspects, including in both the medical and management fields and in economics.

## Figures and Tables

**Table 1:** Manifestation of stress.

Subjective	Objective
Cognitive impairments	Allergic reactions
Tiredness	Eating disorders
Chest pain	Sleep disturbance
Negative impact on knowledge acquisition	
Suspending the selectivity of attention	

Source: [8,9,10].

**Table 2:** Description of the used criteria.

Age	Gender	Education	Profession Type	Dose
Written by the participants by hand	Male	Primary	Medical (doctor, nurse, paramedics, laboratory technicians, medical analysts)	First
	Female	Secondary	Non-medical (others)	Second
		Vocational		
		Technical		
		Post-secondary		
		Higher		

Source: Own study.

**Table 3:** Research results—aggregated form.

List	Goal	N	Test Name	Test Result	Significance (*p*)
1	Normality check for anxiety as state	595	Shapiro–Wilk	0.953	<0.001
2	Normality check for anxiety as trait	595	Shapiro–Wilk	0.939	<0.001
3	Correlation between anxiety as state and anxiety as trait	595	Spearman’s Rho	0.600	<0.001
4	Correlation between age and anxiety as state in the population	595	Spearman’s Rho	0.0333	0.211
5	Correlation between age and anxiety as trait in the population	595	Spearman’s Rho	−0.063	0.062
6	Correlation between age and anxiety as trait for doctors	91	Spearman’s Rho	−0.179	0.045
7	Gender and the anxiety as a state in population	W = 448 M = 147	U Mann–Whitney	U = 25,068.5 z = −4.393	<0.001
8	Gender and the anxiety as a state among medical professions	W = 312 M = 97	U Mann–Whitney	U = 10,048.5 z = −4.005	<0.001
9	Anxiety and education level	Secondary = 128 higher = 405	U Mann–Whitney	U = 22,549 Z = −2.244	0.025
10	Anxiety among medical and non-medical professions	Medical = 409 Non-medical = 186	U Mann–Whitney	U = 33,905.5 z = −2.149	0.032
11	Anxiety among nurses and others	Nurses = 164 Non-medical = 431	U Mann–Whitney	U = 29,461 z = −3.173	0.002
12	Anxiety as a state and the vaccination dose	First dose = 359 Second dose = 431	U Mann–Whitney	U = 41,838.5 Z = −0.258	0.796
13	Anxiety as a trait and the vaccination dose		U Mann–Whitney	U = 41,942 Z = −0.208	0.835

Source: Own study.

## Data Availability

The results are accessible by request due to the ongoing analysis.
